# Resolution Adopted by the Congress on Tuberculosis, New York, November 16, 1906

**Published:** 1906-12

**Authors:** 


Resolution Adopted by the Congress on Tuberculosis, New York, November 16, 1906.—The following resolutions offered by Dr. F. E. Daniel at the 1904 meeting in St. Louis, and unanimously adopted, were reported by the Committee on Resolutions as covering the subject fully and expressing the sense of the Congress. They were unanimously adopted, and a copy ordered to be furnished every delegate from foreign countries:

Whereas, The public health is the paramount interest of every State and Nation, and upon it depend in a measure all other interests; and,

Whereas, In American countries it is less safeguarded than any interests, public, private, personal or property; and,

Whereas, Consumption is the greatest menace and destructive agent to the public health, its mortality, according to the last United States census report being over 400 a day; and,,

Whereas, The cause or causes of this disease, and its methods of dissemination are well understood by medical and sanitary scholars; and,

Whereas, It is known to be an infectious and communicable disease, the prevention of which is within the power and scope of sanitary science; and,

Whereas, It requires the authority of law to institute and enforce the necessary and proper measures of prevention; be it

Resolved, That it is the sense of the American International Congress on Tuberculosis, now in session in New York City, that it is the imperative duty of all civilized governments to take immediate action for the arrest of the spread of this scourge, and, as far as lies within the power of sanitary science and human endeavor, to eradicate it; and further, that it is the sense of this Congress that every government should appoint a Commissioner of the Public Health, with a seat in the Cabinet, and give adequate authority and means to accomplish the desired ends in suppressing tuberculosis; and further, that in each State and Territory of the United States, where there exists a State Board of Health, and in those States where there is no board of health, there should be created a board composed of the ablest sanitarians,—authority should be given to such boards to formulate and enforce a code of regulations for the prevention of the ravages of this fatal scourge.

